# The Severity and Characteristics of Pediatric Group A Streptococcal Meningitis in the Netherlands (2015–June 2024): Results From a Nationwide Multicenter Cohort Study

**DOI:** 10.1097/INF.0000000000005247

**Published:** 2026-04-16

**Authors:** Mirte L. Jansen, Evelien B. van Kempen, Adam J. Tulling, Emilie P. Buddingh’, Nina M. van Sorge, Merijn W. Bijlsma, Mirjam van Veen, on behalf of the COPP-iGAS consortium, Mirjam van Veen

**Affiliations:** Department of Paediatrics, Juliana Children’s Hospital HagaHospital, the Hague, the Netherlands; Department of Paediatrics, Willem-Alexander Children’s Hospital Leiden University Medical Centre, Leiden, the Netherlands; Department of Paediatrics, Juliana Children’s Hospital HagaHospital, the Hague, the Netherlands; Department of Paediatrics, Willem-Alexander Children’s Hospital Leiden University Medical Centre, Leiden, the Netherlands; Department of Paediatrics, Willem-Alexander Children’s Hospital Leiden University Medical Centre, Leiden, the Netherlands; Department of Paediatrics, Albert Schweitzer Hospital, Dordrecht, the Netherlands; Department of Paediatrics, Amsterdam University Medical Centre Emma Children’s Hospital, Amsterdam, the Netherlands; Department of Medical Microbiology and Infection Prevention, Amsterdam University Medical Centre, Amsterdam, the Netherlands; Department of Paediatrics, Bern hoven Hospital, Uden, the Netherlands; Department of Paediatrics, Haaglanden Medical Centre, the Hague, the Nether lands; Department of Paediatrics, Maxima Medical Centre, Veldhoven, the Netherlands; Department of Paediatrics, Radboud University Medical Centre Amalia Children’s Hospital, Nijmegen, the Netherlands; Department of Paediatrics, Northwest Hospital Group, Alkmaar and Den Helder, the Netherlands; Department of Paediatrics, Radboud University Medical Centre Amalia Children’s Hospital, Nijmegen, the Netherlands; National Institute for Public Health and the Environment; Department of Paediatrics, Maasstad Hospital, Rotterdam, the Netherlands; Department of Paediatrics, Division of Pediatric Infectious Diseases and Immunology, Erasmus MC-Sophia Children’s Hospital, University Medical Centre Rotterdam, Rotterdam, the Netherlands; Department of General Paediatrics, Erasmus University Medical Centre Sophia Children’s Hospital, Rotterdam, the Netherlands; Department of Paediatrics, Spaarne Hospital, Haarlem, the Netherlands; Department of Paediatrics, Sint-Franscisus Gasthuis Hospital, Rotterdam, the Netherlands; Department of Paediatrics, Slingeland Hospital, Doetinchem, the Netherlands; Department of Paediatrics, TerGooi Hospital, Hilversum, the Netherlands; Department of Paediatric Infectious Diseases and Immunology, University Medical Centre Utrecht Wilhelmina Children’s Hospital, Utrecht, the Netherlands; Department of Paediatrics/Infectious Diseases, University Medical Centre Groningen Beatrix Children’s Hospital, Groningen, the Netherlands; Department of Paediatrics, Zaans Medical Centre, Zaandam, the Netherlands; From the * Department of Paediatrics, Juliana Children’s Hospital, Haga Hospital, The Hague; † Department of Paediatrics, Willem-Alexander Children’s Hospital, Leiden University Medical Centre, Leiden; ‡ Department of General Paediatrics, Erasmus Medical Centre Sophia, Rotterdam; § Department of Medical Microbiology and Infection Prevention, Netherlands Reference Centre for Bacterial Meningitis, Amsterdam University Medical Centre, University of Amsterdam, Amsterdam; ¶ Department of Paediatrics, Emma Children’s Hospital, Amsterdam University Medical Centre, University of Amsterdam, Amsterdam, the Netherlands.

**Keywords:** *Streptococcus pyogenes*, group A streptococcus, meningitis, pediatrics, epidemiology

## Abstract

**Background:**

From 2022 onwards, several countries, including the Netherlands, reported a marked increase in invasive group A streptococcal (iGAS) infections. Therefore, we aimed to describe the clinical presentation, disease course, treatment and outcomes of group A streptococcus (GAS) meningitis, a rare but severe manifestation of GAS infection in pediatric patients.

**Methods:**

Children with GAS meningitis were selected from the COPP-iGAS study, a national observational cohort study in children 0–17 years of age with in-hospital diagnosis of iGAS between 2015 and June 2024, conducted across 20 hospitals in the Netherlands, including all 7 academic centers with a pediatric intensive care unit.

**Results:**

Twenty-seven children were included, of whom 41% (11/27) were younger than 5 years of age. Most patients presented during the first quarter of the year (n = 13/27, 48%). Admission to an intensive care unit occurred in 15/27 (56%) patients and 4/27 (15%) patients died. Detailed clinical data were available for 13/27 patients. Clinical course data could be evaluated for 12 of these patients. Among those 12 patients, almost two-thirds had a complicated disease course, mainly involving cardiorespiratory failure (4/12, 33%). All 12 patients received prolonged antibiotic therapy [median 43 days (IQR 14–61)], predominantly intravenously [median 42 days (IQR 14–50)]. Adjunctive therapy included corticosteroids (6/12, 50%), antiviral agents (3/12, 25%) and anticoagulants (3/12, 25%).

**Conclusion:**

Our findings highlight that pediatric GAS meningitis is a severe condition characterized by high intensive care admission rates and substantial mortality. In the subgroup with detailed clinical data, complications were frequent and prolonged antibiotic treatment was common. Increased clinical awareness is warranted.

*Streptococcus pyogenes*, or group A streptococcus (GAS), is a Gram-positive bacterium that colonizes the skin and naso- and oro-pharynx but can also cause a wide spectrum of infections.^1^ Noninvasive GAS infections, such as impetigo, scarlet fever and pharyngitis, are typically mild and self-limiting. In contrast, invasive GAS (iGAS) infections are associated with severe and potentially life-threatening diseases, including sepsis, meningitis, necrotizing fasciitis and streptococcal toxic shock syndrome.

In the post-COVID-19 pandemic era, a worldwide surge in pediatric iGAS infections has been observed.^2–11^ In the Netherlands, an almost 3-fold increase in pediatric iGAS cases was seen compared to the pre-COVID-19 period.^2,12^ This increase was likely multifactorial, driven by a postpandemic increase in cocirculating respiratory viruses, emergence of more virulent GAS strains and the concept of “immunity debt” resulting from reduced exposure to infectious diseases during COVID-19–related public health measures.^3,13–17^ GAS meningitis, one of the rare manifestations of iGAS, even rose by 12.3-fold in the Netherlands following the COVID-19 pandemic.^2^ Importantly, it was associated with an increased risk on intensive care unit (ICU) admission and death, as compared to all iGAS infections.^2^ Interestingly, not all countries experiencing a rise in iGAS have observed a similar increase in GAS meningitis cases.^5^ The reason for this discrepancy remains unclear. In light of our recent experience with an increase in GAS meningitis cases, there is a pressing need to improve our understanding of this disease. The aim of this study was to describe the clinical presentation, disease course, treatment and outcomes of pediatric GAS meningitis, to improve understanding and increase clinical awareness of this rare but severe condition.

## METHODS

### Study Design

This is a subgroup analysis of the Dutch COPP-iGAS study, a national multicenter observational cohort study conducted across 20 hospitals, including all 7 Dutch academic hospitals with pediatric intensive care unit, focusing on pediatric iGAS infections.^2^ In the COPP-iGAS study, children 0–17 years of age with an in-hospital diagnosis of iGAS infection [at emergency department presentation, hospitalization, or postmortem, including deaths before hospital arrival], between January 2015 and June 2024, were included. Patients were enrolled retrospectively from January 2015 to December 2021 and prospectively from January 2022 up to July 2024. For prospectively enrolled patients, informed consent was obtained, allowing detailed clinical data collection. For patients without informed consent (ie, those diagnosed during the retrospective period and those diagnosed prospectively but who could not be traced), concise data were collected under a waiver. Availability of detailed clinical data was therefore determined by informed consent during the prospective study period rather than by hospital type.

The study periods were defined as: pre-COVID-19 period (January 2015 to March 2020), the COVID-19 period (April 2020 to December 2021) and the post-COVID-19 period (January 2022 up to June 2024). The study method has previously been published.^2^

### Study Population

In the COPP-iGAS study, an iGAS infection was defined as (1) positive culture or molecular detection (polymerase chain reaction–based methods) of GAS in a normally sterile body site such as blood, cerebrospinal fluid (CSF), pleural fluid, or synovial fluid; (2) clinical diagnosis of streptococcal toxic shock syndrome or necrotizing fasciitis (with or without microbiologic confirmation); or (3) positive molecular detection or culture of GAS from a nonsterile body site with clinical features compatible with iGAS with no other causative microorganism isolated.^2^ Patients were identified retrospectively via the hospital’s microbiology databases and/or prospectively by local investigators. For this subgroup analysis, patients diagnosed with GAS meningitis were selected. GAS meningitis was defined as (1) patients with clinical symptoms (fever, headache, neck stiffness, vomiting, petechiae, or altered mental status) and laboratory findings (increased C-reactive protein, abnormal CSF values), suggestive of meningitis according to the treating physician plus (2) microbiologic confirmation by a positive culture or molecular detection for GAS from a sterile site such as blood or CSF, or from a nonsterile site such as the ear, nasopharynx, or skin. In contradiction to the COPP-iGAS consortium, 1 additional participant was included whose clinical course occurred within the study period, though data collection was finalized just after the study period (July 2024).

### Data Collection

GAS meningitis cases were identified though the COPP-iGAS study database.^2^ Detailed data collection included medical history, medical care encounters preceding 14 days of hospitalization, admission duration, clinical characteristics, laboratory results, clinical course, treatments, interventions and outcome at discharge. Severe disease was defined as the need for ICU admission or death. Predisposing conditions included: prior upper respiratory tract infections, varicella zoster virus infections, parameningeal infections (involving sinusitis, otitis media and mastoiditis), soft-tissue infections (involving necrotizing fasciitis, cellulitis, erysipelas and myositis) and anatomical factors. *emm*-typing was obtained from electronic health records or through probabilistic linkage to data from the National Reference Laboratory for Bacterial Meningitis (NRLBM, Amsterdam UMC, Netherlands) database, as described for the COPP-iGAS study.^2^ Concise data included age (stratified as <5 years, 5–9 years and >10 years), month and year of iGAS diagnosis, predisposing infections (limited to signs of respiratory tract infection and skin infections), and outcome.

### Analysis

Descriptive and statistical analysis were performed using IBM SPSS Statistics (SPSS Statistics for Windows, Version 28.0, IBM Corp, Armonk, NY). Continuous data were reported as median with interquartile ranges (IQR) if data were not normally distributed. Normality was assessed using the Kolmogorov-Smirnov test. Categorical data were presented as counts and percentages, and comparisons were performed using χ^2^ and Fisher exact tests, as appropriate.

### Ethical Approval

The COPP-iGAS study was approved by the medical ethical review committee (METC-LDD N20.043). Informed consent was waived for concise retrospective data collection, while informed consent was obtained for prospective cases with extensive data collection. Study protocols are available on the study website (www.infectiekids.nl).

## RESULTS

### Cohort Description

**FIGURE 1.**
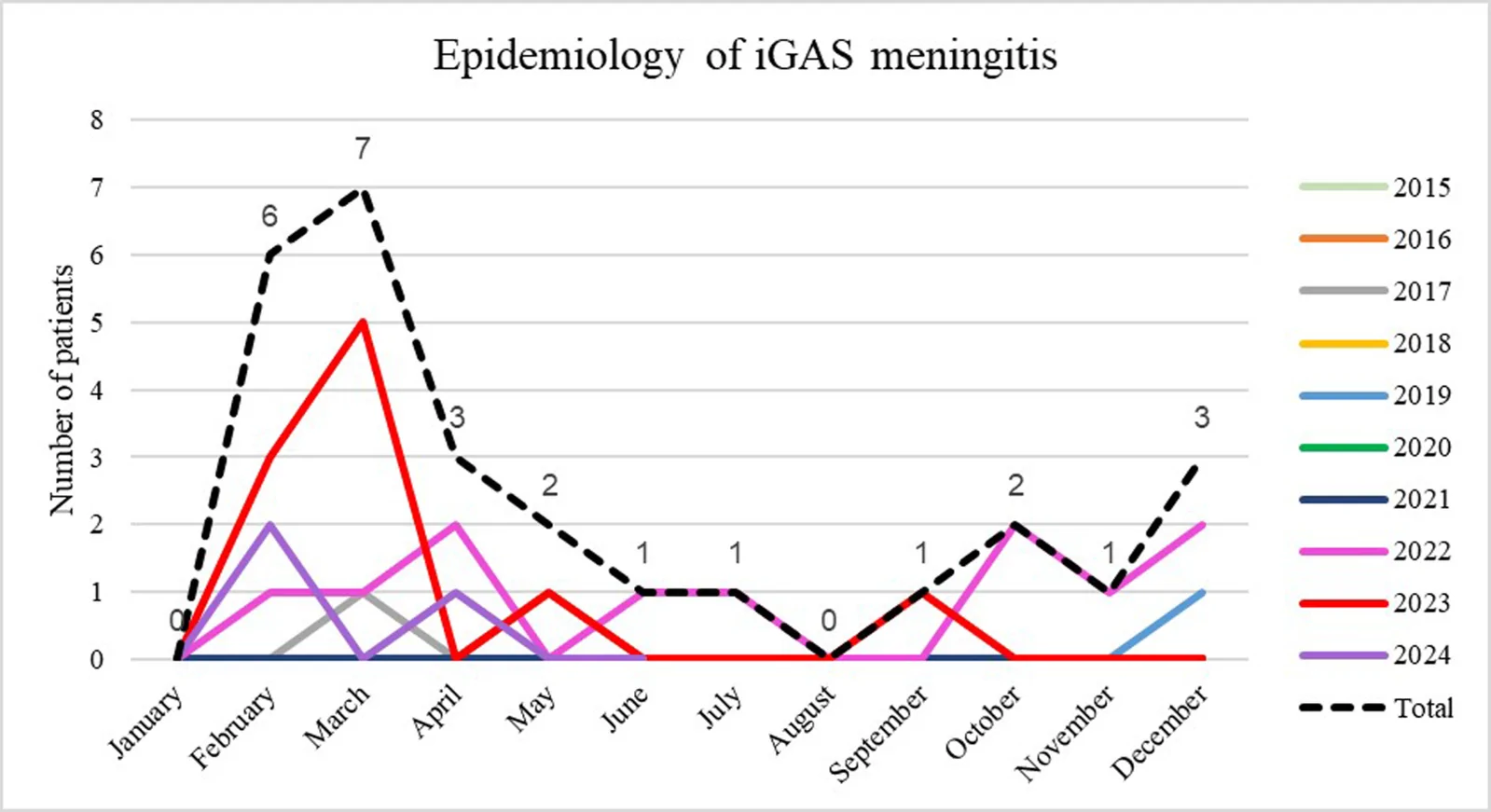
Number of pediatric GAS meningitis cases displayed per month between 2015 and June 2024.

A total of 27 (4.2%) children with GAS meningitis were identified, representing 4.2% of the 645 children with an iGAS infection (Figure, Supplemental Digital Content 1, https://links.lww.com/INF/G604). Detailed data were available for 13 patients. Across the study period, GAS meningitis occurred most frequently in the first quarter of the year (Q1; n = 13/27, 48%), as visualized in Figure 1. Patient characteristics are described in Table 1. 41% (11/27) of patients were younger than 5 years of age. Among the 13 patients with detailed data available, the median age was 5.6 years (IQR 3.3–8.2) and 46% (6/13) were male. When considering all 27 cases, the age distribution differed between the pre-COVID-19 and post-COVID-19 periods: in the pre-COVID-19 cohort, all patients (3/3, 100%) were older than 10 years, compared with 17% (14/27) in the post-COVID-19 cohort (*P* = 0.008). In the 2 weeks preceding hospitalization, 14/27 (52%) had a preceding infection, including respiratory tract infections (9/27, 33%) or a predisposing skin infection (6/27, 22%); 3 (3/27, 11%) of the skin infections were caused by varicella zoster. Among the 13 patients with detailed data, additional predisposing conditions were parameningeal infection in 3 patients (23%), and an auditory brain implant in 1 patient. 15% (4/27) of patients died and 56% (15/27) required ICU admission. Of the 4 deaths, 2 occurred before hospitalization.

**TABLE 1.** Characteristics of Patients With Pediatric Group A Streptococcus Meningitis, Full Cohort

Characteristics	2015–March 2020 n = 3	2022–June 2024 n = 24	Full Cohort n = 27	*P*-value
Age (years, stratified), no. (%)				0.008
0–4	0	11 (46)	11 (41)	
5–9	0	9 (38)	9 (33)	
10–17	3 (100)	4 (17)	7 (26)	
Predisposing conditions, no. (%)	2 (67)	12 (50)	14 (52)	0.529*
Upper respiratory tract infection	1 (33)	8 (33)	9 (33)	0.721*
Skin infection†	1 (33)	5 (21)	6 (22)	0.545*
Varicella-zoster infection	0	3 (11)	3 (11)	
ICU admission	1 (33)	14 (58)	15 (56)	0.414*
Mortality	0	4 (17)	4 (15)	0.605*

* Fisher exact test.

† Three patients with varicella-zoster, 1 with scarlet fever, 1 with exanthema/swelling at the shoulder and 2 patients were not further specified.

### Clinical Presentation

**FIGURE 2.**
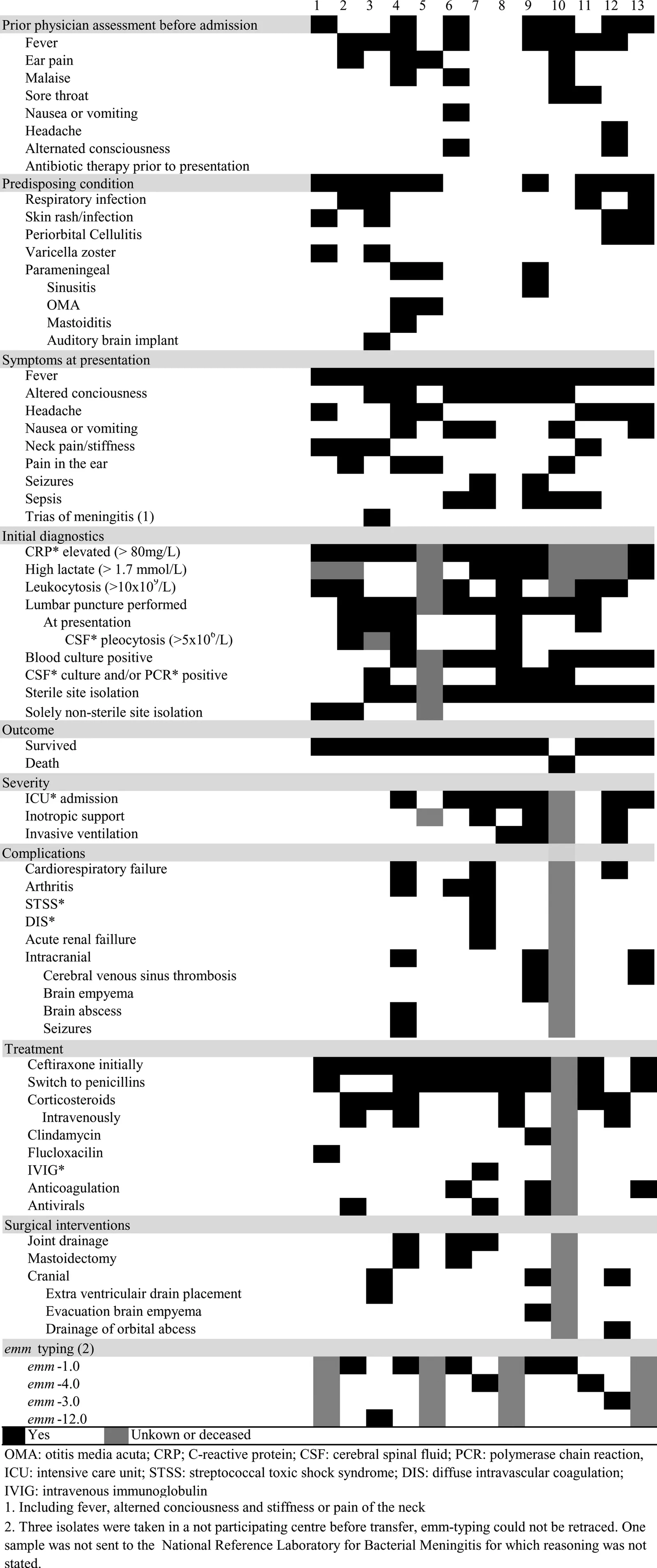
Heatmap illustrating clinical features of a patient with detailed data.

Among the 13 patients with detailed data, the median duration of symptoms before hospitalization was 4 days (IQR 1–5) (Table, Supplemental Digital Content 2, https://links.lww.com/INF/G604). The main complaints reported at home were fever (8/13, 61.5%), symptoms of respiratory tract infection (7/13, 53.8%) and general malaise (3/13, 23.1%) (Fig. 2). 7/13 (54%) patients had been assessed by a physician before hospital admission. At presentation, fever was present in all patients (13/13, 100%), while altered consciousness (7/13, 54%), headache (6/13, 46%) and nausea or vomiting (5/13, 38%) were also common (Fig. 2). Neck stiffness or pain was documented under “other complaints” in 4 patients (31%), one of whom presented with the classic triad of meningitis. Presentation was heterogeneous, with some children presenting predominantly with meningeal features and others with more systemic illness. C-reactive protein was measured at presentation in 9 patients (69%) and was elevated in all, with a median value of 223 mg/dL (IQR 170–310). A lumbar puncture was performed in 61% (8/13) patients, of which 5/13 (38%) at presentation. Available CSF findings are summarized in the Table, Supplemental Digital Content 2, https://links.lww.com/INF/G604.

### Clinical Course, Treatment and Outcome

Among the 13 patients with detailed data, 1 patient died shortly after arrival at the emergency department. Clinical course and in-hospital treatment could therefore be evaluated and presented for 12 patients. The median length of stay was 9 days (IQR 4–19). Among the 7/12 (58%) patients with ICU admission, the median duration of stay was 1 day (IQR 1–4). Complications were observed in 7/12 (58%) patients, including cardiorespiratory failure in 4 patients (33%) and arthritis in 3 patients (25%), one of whom additionally developed streptococcal toxic shock syndrome, disseminated intravascular coagulation and renal failure. Intracranial complications occurred in 3/12 (25%), including cerebral venous thrombosis in 2 patients (17%), one of whom also had a brain empyema, and a brain abscess with seizures in the third patient. Surgical interventions involved joint drainage for arthritis (3/12, 25%), mastoidectomy (2/12, 17%), evacuation of brain empyema (1/12, 8%), evacuation of orbital abscess (1/12, 8%) and placement of an external ventricular drain (1/12, 8%). All 12 patients received antibiotic therapy. At presentation, 11/12 (92%) patients were administered ceftriaxone. Antibiotic regimens were subsequently modified in 11/12 (92%) patients, mainly to penicillin to narrow the spectrum based on microbiological results (9/11, 82%). One (8%) patient initially received amoxicillin-clavulanic acid, but was switched to ceftriaxone the following day because of clinical deterioration. Additional therapies included metronidazole in 3/12 patients (25%), flucloxacillin in 1/12 (8%) and clindamycin in 1/12 (8%). The median total duration of antibiotic therapy was 43 days (IQR 14–61), with a median intravenous treatment duration of 42 days (IQR 14–50). In addition, corticosteroids were administered to 6/12 (50%) patients, of whom 4 (33%) received them intravenously and antiviral therapy in 3/12 (25%). Intravenous immunoglobulins were given to 1 patient (8%) with streptococcal toxic shock syndrome. Anticoagulants were administered to 2 patients (17%) with cerebral venous sinus thrombosis and 1 patient (8%) for an unspecified indication. One patient (8%) received antiepileptic treatment because of seizures. Three of 12 patients received inotropic treatment (25%).

### Microbiology

Among the 13 children with detailed data, GAS was isolated from a sterile site in 10 cases, most commonly from blood cultures (8/13, 61.5%), and from a nonsterile site in 2 cases in combination with clinical features consistent with iGAS; in 1 case, the site of isolation was not specified because of missing data. *emm*-typing was available for isolates of 9/13 (69%) patients. The most common type was *emm*-type 1.0 (5/9, 56%), followed by *emm*-type 4.0 (2/9, 22%), *emm*-type 12.0 (1/9, 11%) and *emm*-type 3.93 (1/9, 11%).

## DISCUSSION

Following the 12-fold post-COVID-19 increase in pediatric GAS meningitis in the Netherlands, this subgroup analysis highlights the severity of the disease. More than half of the patients required ICU admission and 15% died. These findings are consistent with previously reported ICU admission rates of 47%–50% and mortality rates of 16%–17%.^18,19^ Among the patients with detailed data collection, complications were frequent, prolonged antibiotic treatment was common, and half underwent surgical intervention, further underscoring the clinical severity of pediatric GAS meningitis.

Among the 12 patients with data on clinical course, the median duration of antibiotic therapy was 43 days, considerably longer than the 18 days reported by Di Meglio et al and the 7–21 days typically recommended for bacterial meningitis caused by other pathogens.^19,20^ The exact rationale for the prolonged duration of antibiotic therapy could not be determined from the available data. However, this may reflect the severity of disease, the relatively high number of surgical interventions, and a cautious approach by treating clinicians in the absence of cohort studies to guide treatment duration in pediatric GAS meningitis. The types of complications reported among those 12 patients, including cardiorespiratory failure, arthritis and cerebral venous sinus thrombosis, were like those reported in previous studies.^18–20^ The higher rate of surgical intervention in our cohort compared with earlier reports (50% vs. 23% in Di Meglio et al and 39% in Dou et al) may partly be explained by our inclusion of joint drainage procedures.^18,19^

Among the patients with detailed data, GAS was identified in blood cultures in two-thirds of cases. This may partly be explained by the relatively low proportion of lumbar punctures performed at presentation (42%) and by reduced CSF culture yield after prompt initiation of antibiotics. *emm* typing was available for most isolates in this subgroup, with *emm*-type 1.0 predominating. This proportion was slightly higher than in the overall COPP-iGAS cohort (56% vs. 40.6%, respectively).^2^ Previous studies have likewise reported predominance of *emm*-type 1.0 in central nervous system GAS infections.^5,15,21–24^ A new variant of *emm*-type 1.0, M1_UK,_ dominated 88% of iGAS strains during 2022–2023 in Dutch adults with GAS meningitis and was associated with a higher case fatality rate.^23^ Because whole-genome sequencing was not performed in our study, the presence of M1UK could not be assessed.

At the cohort level, GAS meningitis occurred most frequently in the first quarter of the year, aligning with seasonal peaks of iGAS and high circulation of viruses such as influenza and varicella. Moreover, a probable source of infection was identified in two-third of patients, mainly the upper respiratory tract, ear-nose-throat (ENT) and skin infections, consistent with previous studies.^14,18,19,22^ Varicella and upper respiratory tract infections have been linked to iGAS, while ENT and craniofacial skin infections appear to predispose to parameningeal spread, as is observed in our population.^11,18,19,21,22,25,26^

Pediatric GAS meningitis remains rare in the broader context of bacterial meningitis. In 2024 the National Reference Laboratory for Bacterial Meningitis, which receives approximately 90% of bacterial meningitis isolates in the Netherlands, reported that bacterial meningitis in pediatric patients (0–19 years) was caused by *S. pyogenes* in 2.9% of cases.^27^ Although comparative pediatric data are limited, the mortality observed in our cohort was like previously reported mortality in pediatric bacterial meningitis overall (14.8%; 1935–2019).^28^ The proportion of pediatric meningitis patients requiring ICU admission is unknown, although a Dutch study in patients >16 years reported an ICU admission rate of 51% (2006–2022).^29^ Among the patients with detailed data, the initial presentation was heterogeneous. While some children presented with features suggestive of meningitis, others had a more systemic presentation with central nervous system involvement. Previous studies have also shown that pediatric GAS meningitis often arises through hematogenous spread or by continuous spread from extracranial foci, particularly ear, sinus, or skin infections. The presence of systemic involvement is shown to be common and associated with poor outcome.^18,19^ Among the patients with detailed data, the presenting symptoms of GAS meningitis overlapped with those of other bacterial meningitides, particularly fever and, in a subset of patients, altered consciousness, headache, nausea or vomiting and neck pain or stiffness, whereas di Meglio et al reported fever and meningeal signs in all patients.^19^ Inflammatory markers, however, were consistently elevated in patients with available laboratory data. These findings suggest that GAS meningitis should be considered in children with (persistent) fever, neurological symptoms and elevated inflammatory markers, particularly in the context of preceding upper respiratory tract, ENT, or craniofacial infections, even when the initial presentation is dominated by sepsis. As these features are not specific to GAS, distinguishing GAS meningitis from other acute bacterial meningitides or invasive bacterial infections at initial presentation remains difficult, also highlighted by Dou et al and Meglio et al.^18,19^ This heterogeneity may be clinically relevant, as meningitis may be less readily suspected when the initial presentation is dominated by sepsis or shock. Data on the timing of clinical suspicion of central nervous system infection were not systematically collected and could therefore not be analyzed. Given the small number of patients and incomplete CSF data, we could not reliably distinguish phenotypic subgroups or assess whether CSF findings differed between these presentations. Evidence for adjunctive corticosteroid therapy in bacterial meningitis is derived mainly from studies of acute bacterial meningitis overall, rather than GAS meningitis specifically. Although the clearest pathogen-specific benefit in children has been reported for *Haemophilus influenzae* meningitis, broader pediatric evidence also suggests benefit with respect to hearing loss and neurological sequelae.^30,31^ Evidence for *S. pyogenes* meningitis specifically is lacking, and a pathogen-specific randomized trial is unlikely to be feasible. Nevertheless, Dutch guidelines recommend adjunctive dexamethasone for all patients older than 1 month presenting with bacterial meningitis, since at presentation, the causal pathogen is still unclear. The observed variation in corticosteroid use in our cohort may therefore reflect variation in adherence to the guideline in clinical practice, as well as uncertainty when the initial presentation was more suggestive of sepsis than meningitis.^30^

### Strengths and Limitations

This study has several strengths. Our study used consistently collected data from one of the largest pediatric iGAS cohorts, drawn from academic referral centers, teaching hospitals and regional hospitals. This comprehensive clinical information gathered in the past 10 years provides valuable insights into clinical characteristics, disease severity, outcomes and management. Furthermore, given the limited number of cohort studies on pediatric GAS meningitis, our study represents an important contribution to the literature and helps define the clinical spectrum of this rare but severe disease.

Some limitations must be acknowledged. First, the small sample size limits the statistical power and generalizability. However, given the rarity of GAS meningitis, nationwide data are unlikely to yield substantially larger cohorts. Second, predisposing respiratory or skin infections may be underreported as the diagnoses relied on clinical assessment. However, this approach likely captures clinically relevant infections more comprehensively than microbiology alone, as it is not routinely obtained. Third, because the cohort included both retrospectively and prospectively enrolled patients, detailed clinical data were available for only a subgroup of patients. As a result, clinical presentation, treatment, complications and microbiological findings could not be described for the full cohort of 27 children, which may have introduced information bias. Despite these limitations, our findings have important clinical and research implications. We emphasized the severity of pediatric GAS meningitis and the need for heightened clinical awareness during iGAS upsurges. Clinicians should maintain a high index of suspicion for GAS meningitis, particularly in young children with preceding upper respiratory tract, ENT, or craniofacial infections and during periods of high iGAS activity, as we encountered in 2022–2023. Even though GAS meningitis is rare, our study highlights the need for larger, international studies to further improve early recognition and guide management strategies, including the optimal duration of antibiotic therapy and the role of adjunctive treatments. Collectively, these efforts may inform evidence-based guidelines and improve the management of pediatric GAS meningitis in the future.

## CONCLUSION

Our findings emphasize the severity of pediatric GAS meningitis, characterized by high rates of ICU admission, mortality, disease complications and surgical interventions. The recent post-COVID-19 surge in GAS meningitis cases highlights the need for heightened clinical vigilance and prompt management when suspected to possibly improve. Clinicians should consider GAS meningitis in children with persistent fever, neurological symptoms and elevated inflammatory markers, particularly when preceded by upper respiratory tract, ENT, or craniofacial infections. Careful monitoring for complications remains essential to improve outcomes in this rare but life-threatening condition.

The members of the COPP-iGAS collaborative include: Mirjam van Veen (Department of Paediatrics, Juliana Children’s Hospital HagaHospital, the Hague, the Netherlands), Emmeline Buddingh (Department of Paediatrics, Willem-Alexander Children’s Hospital Leiden University Medical Centre, Leiden, the Netherlands), Evelien van Kempen (Department of Paediatrics, Juliana Children’s Hospital HagaHospital, the Hague, the Netherlands), Adam Tulling (Department of Paediatrics, Willem-Alexander Children’s Hospital Leiden University Medical Centre, Leiden, the Netherlands), Erik von Asmuth (Department of Paediatrics, Willem-Alexander Children’s Hospital Leiden University Medical Centre, Leiden, the Netherlands), Ankie Lebon (Department of Paediatrics, Albert Schweitzer Hospital, Dordrecht, the Netherlands), Merijn Bijlsma (Department of Paediatrics, Amsterdam University Medical Centre Emma Children’s Hospital, Amsterdam, the Netherlands), Nina van Sorge (Department of Medical Microbiology and Infection Prevention, Amsterdam University Medical Centre, Amsterdam, the Netherlands), Jan van der Linden (Department of Paediatrics, Bern hoven Hospital, Uden, the Netherlands), Jantien Bolt (Department of Paediatrics, Haaglanden Medical Centre, the Hague, the Nether lands), Lonneke van Onzenoort-Bokken (Department of Paediatrics, Maxima Medical Centre, Veldhoven, the Netherlands), Else Bijker (Department of Paediatrics, Radboud University Medical Centre Amalia Children’s Hospital, Nijmegen, the Netherlands), Dorine Borensztajn (Department of Paediatrics, Northwest Hospital Group, Alkmaar and Den Helder, the Netherlands), Kim Stol (Department of Paediatrics, Radboud University Medical Centre Amalia Children’s Hospital, Nijmegen, the Netherlands), Brechje de Gier (National Institute for Public Health and the Environment), Navin Boeddha (Department of Paediatrics, Maasstad Hospital, Rotterdam, the Netherlands; Department of Paediatrics, Division of Pediatric Infectious Diseases and Immunology, Erasmus MC-Sophia Children’s Hospital, University Medical Centre Rotterdam, Rotterdam, the Netherlands), Rianne Oostenbrink 4 (Department of General Paediatrics, Erasmus University Medical Centre Sophia Children’s Hospital, Rotterdam, the Netherlands), Marlies van Houten (Department of Paediatrics, Spaarne Hospital, Haarlem, the Netherlands), Gerdien Tramper (Department of Paediatrics, Sint-Franscisus Gasthuis Hospital, Rotterdam, the Netherlands), Monique Jacobs (Department of Paediatrics, Slingeland Hospital, Doetinchem, the Netherlands), Caroline KosterinkBrackel (Department of Paediatrics, TerGooi Hospital, Hilversum, the Netherlands), Joanne Wildenbeest (Department of Paediatric Infectious Diseases and Immunology, University Medical Centre Utrecht Wilhelmina Children’s Hospital, Utrecht, the Netherlands), Aline Verhage (Department of Paediatrics/Infectious Diseases, University Medical Centre Groningen Beatrix Children’s Hospital, Groningen, the Netherlands) and Leontien van der Aa (Department of Paediatrics, Zaans Medical Centre, Zaandam, the Netherlands).

## ACKNOWLEDGMENTS

*Members of the COPP-IGAS consortium are listed in Supplemental Digital Content 3, https://links.lww.com/INF/G604*.

## Supplementary Material


