# Supplementary material for: The Severity and Characteristics of Pediatric Group A Streptococcal Meningitis in the Netherlands (2015–June 2024): Results From a Nationwide Multicenter Cohort Study
Source: Pediatr Infect Dis J. 2026 Apr 16;45(10):859–64. doi: 10.1097/INF.0000000000005247 (PMC13557488; doi:10.1097/INF.0000000000005247)
Supplement: Supplementary file 1 [file inf-45-0859-s001.pdf]

SUPPLEMENTAL DIGITAL CONTENT 1. Flowchart study population

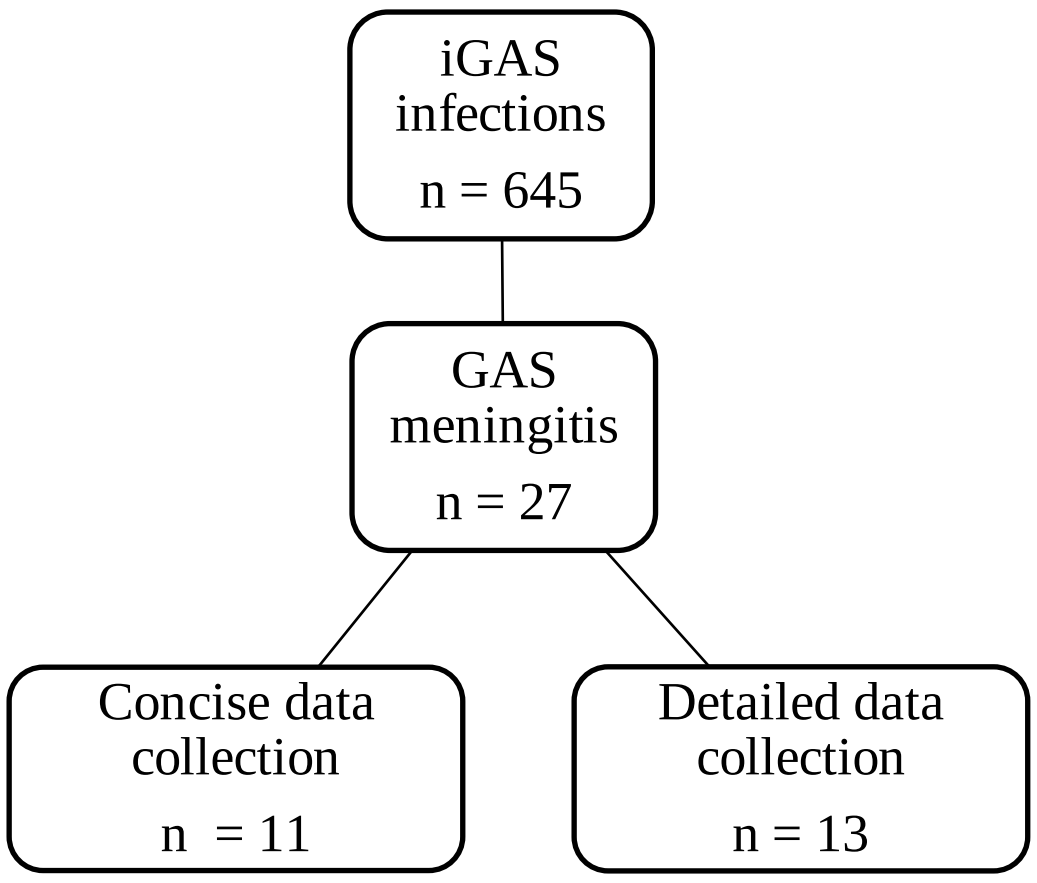

SUPPLEMENTAL DIGITAL CONTENT 2. Non-binary data of detailed cohort of outcome and laboratory parameters

	Total
Length of hospital stay (days), median (IQR) (n = 12)	9 [4 - 19]
Length of ICU stay (days), median (IQR) (n = 8)	1 [1 - 4]
Duration antibiotic therapy (days), median (IQR) (n = 10)	43 [14 - 61]
Intravenous (days), median (IQR) (n = 10)	42 [14 - 50]
Laboratory parameters at presentation	
Leukocytes, median (IQR) (n = 11)	15.5 [9.1-32.1]
CRP (mg/dL), median (IQR) (n = 9)	223 [171-310]
Lactate (mmol/L), median (IQR) (n = 7)	2.5 [1.9-4.7]
Natrium (mmol/L), median (IQR) (n = 9)	130 [127 – 136]

CRP: C-reactive protein; CSF: cerebral spinal fluid

SUPPLEMENTAL DIGITAL CONTENT 3. Members of COPP-iGAS consortium

The members of the COPP-iGAS collaborative include: Mirjam van Veen (Department of Paediatrics, Juliana Children's Hospital HagaHospital, the Hague, the Netherlands), Emmeline Buddingh (Department of Paediatrics, Willem-Alexander Children's Hospital Leiden University Medical Centre, Leiden, the Netherlands), Evelien van Kempen (Department of Paediatrics, Juliana Children's Hospital HagaHospital, the Hague, the Netherlands), Adam Tulling (Department of Paediatrics, Willem-Alexander Children's Hospital Leiden University Medical Centre, Leiden, the Netherlands), Erik von Asmuth (Department of Paediatrics, Willem-Alexander Children's Hospital Leiden University Medical Centre, Leiden, the Netherlands), Ankie Lebon (Department of Paediatrics, Albert Schweitzer Hospital, Dordrecht, the Netherlands), Merijn Bijlsma (Department of Paediatrics, Amsterdam University Medical Centre Emma Children's Hospital, Amsterdam, the Netherlands), Nina van Sorge (Department of Medical Microbiology and Infection Prevention, Amsterdam University Medical Centre, Amsterdam, the Netherlands), Jan van der Linden (Department of Paediatrics, Bern hoven Hospital, Uden, the Netherlands), Jantien Bolt (Department of Paediatrics, Haaglanden Medical Centre, the Hague, the Nether lands), Lonneke van Onzenoort-Bokken (Department of Paediatrics, Maxima Medical Centre, Veldhoven, the Netherlands), Else Bijker (Department of Paediatrics, Radboud University Medical Centre Amalia Children's Hospital, Nijmegen, the Netherlands), Dorine Borensztajn (Department of Paediatrics, Northwest Hospital Group, Alkmaar and Den Helder, the Netherlands), Kim Stol (Department of Paediatrics, Radboud University Medical Centre Amalia Children's Hospital, Nijmegen, the Netherlands), Brechje de Gier (National Institute for Public Health and the Environment), Navin Boeddha (Department of Paediatrics, Maasstad Hospital, Rotterdam, the Netherlands; Department of Paediatrics, Division of Pediatric Infectious Diseases and Immunology, Erasmus MC-Sophia Children's Hospital,

University Medical Centre Rotterdam, Rotterdam, the Netherlands), Rianne Oostenbrink (Department of General Paediatrics, Erasmus University Medical Centre Sophia Children's Hospital, Rotterdam, the Netherlands), Marlies van Houten (Department of Paediatrics, Spaarne Hospital, Haarlem, the Netherlands), Gerdien Tramper (Department of Paediatrics, Sint-Franscisus Gasthuis Hospital, Rotterdam, the Netherlands), Monique Jacobs (Department of Paediatrics, Slingeland Hospital, Doetinchem, the Netherlands), Caroline Kosterink-Brackel (Department of Paediatrics, TerGooi Hospital, Hilversum, the Netherlands), Joanne Wildenbeest (Department of Paediatric Infectious Diseases and Immunology, University Medical Centre Utrecht Wilhelmina Children's Hospital, Utrecht, the Netherlands), Aline Verhage (Department of Paediatrics/Infectious Diseases, University Medical Centre Groningen Beatrix Children's Hospital, Groningen, the Netherlands) and Leontien van der Aa (Department of Paediatrics, Zaans Medical Centre, Zaandam, the Netherlands).
